# Phylogenetic radiation of the greenbottle flies (Diptera, Calliphoridae, Luciliinae)

**DOI:** 10.3897/zookeys.568.6696

**Published:** 2016-02-23

**Authors:** Kirstin A. Williams, Jennifer Lamb, Martin H. Villet

**Affiliations:** 1 Entomology Department, Durban Natural Science Museum, Durban, South Africa; 2 Southern African Forensic Entomology Research Laboratory, Department of Zoology and Entomology, Rhodes University, Grahamstown, South Africa; 3 School of Life Sciences, University of KwaZulu-Natal, South Africa

**Keywords:** *Lucilia sericata*, *Lucilia cuprina*, molecular systematics, parasitism, myiasis

## Abstract

The subfamily Luciliinae is diverse and geographically widespread. Its four currently recognised genera (*Dyscritomyia* Grimshaw, 1901, *Hemipyrellia* Townsend, 1918, *Hypopygiopsis* Townsend 1916 and *Lucilia* Robineau-Desvoidy, 1830) contain species that range from saprophages to obligate parasites, but their pattern of phylogenetic diversification is unclear. The *28S rRNA*, *COI* and *Period* genes of 14 species of *Lucilia* and *Hemipyrellia* were partially sequenced and analysed together with sequences of 11 further species from public databases. The molecular data confirmed molecular paraphyly in three species-pairs in *Lucilia* that hamper barcode identifications of those six species. *Lucilia sericata* and *Lucilia cuprina* were confirmed as mutual sister species. The placements of *Dyscritomyia* and *Hypopygiopsis* were ambiguous, since both made *Lucilia* paraphyletic in some analyses. Recognising *Hemipyrellia* as a genus consistently left *Lucilia* s.l. paraphyletic, and the occasionally-recognised (sub)genus *Phaenicia* was consistently paraphyletic, so these taxa should be synonymised with *Lucilia* to maintain monophyly. Analysis of a matrix of 14 morphological characters scored for adults of all genera and for most of the species included in the molecular analysis confirmed several of these findings. The different degrees of parasitism were phylogenetically clustered within this genus but did not form a graded series of evolutionary stages, and there was no particular relationship between feeding habits and biogeography. Because of the ubiquity of hybridization, introgression and incomplete lineage sorting in blow flies, we recommend that using a combination of mitochondrial and nuclear markers should be a procedural standard for medico-criminal forensic identifications of insects.

## Introduction

All four genera of the subfamily Luciliinae are reported to exhibit parasitism in the form of myiasis – the infestation of humans’ and other animals’ living tissues by fly larvae (Stevens 2003) – ranging from facultative secondary necrophagous myiasis in species like *Lucilia sericata* (Meigen, 1826) to obligate primary carnivorous myiasis in species such as *Lucilia bufonivora* Moniez, 1876. *Lucilia cuprina* (Wiedemann, 1830) and *Lucilia sericata* are noted veterinary pests. Molecular approaches to the management of these flies’ populations can be built on a phylogenetic analysis of the species, but such analyses based on morphological data (Stevens and Wall 1997, Otranto and Stevens 2002, Stevens 2003) have found no evolutionary pattern underlying the radiation of feeding behaviours in *Lucilia* Robineau-Desvoidy, 1830, and biogeographical patterns in the different forms of myiasis have yet to be studied. Furthermore, several taxonomic questions remain regarding the subfamily, from the molecular identification of its species to the definitions of its genera.

At the highest taxonomic level, Rognes (1991) suggested that the genera *Dyscritomyia* Grimshaw, 1901, *Hemipyrellia* Townsend, 1918, *Hypopygiopsis* Townsend 1916, and *Lucilia* Robineau-Desvoidy, 1830 should be united in the subfamily Luciliinae. Several phylogenetic studies have placed species of *Hemipyrellia* within *Lucilia* (Wells et al. 2007, Park et al. 2009, Liu et al. 2011, McDonagh and Stevens 2011). Evidence of whether *Dyscritomyia* is related to *Lucilia* or nested within it has depended on which gene was analysed (Wells et al. 2007, McDonagh and Stevens 2011). The definitions and relationships of these genera therefore need attention.

Several other genera have been included in the Luciliinae, such as *Bufolucilia* Townsend, 1919, *Francilia* Shannon, 1924, *Acrophagella* Ringdahl, 1942, *Phumonesia* Villeneuve, 1914 and *Viridinsula* Shannon, 1926 but most of these are now treated as synonyms of *Lucilia*. *Lucilia* itself has been variously divided into subgenera (Malloch 1926) or genera (Hall 1948), respectively. *Phaenicia* Robineau-Desvoidy, 1863 has been the most used of these names and its use persists (e.g. Park et al. 2009) even though its validity has been challenged regularly (Aubertin 1933, Zumpt 1965, Stevens and Wall 1996). A phylogenetic study of *Lucilia* presents an opportunity to assess this matter.

The largest genus in the subfamily, *Lucilia* has received few quantitative phylogenetic studies (Aubertin 1933, Stevens and Wall 1996, 1997, Wells et al. 2007, Park et al. 2009, DeBry et al. 2012, Sonet et al. 2012), with research generally focusing on species of medical, veterinary or forensic interest in specific geographic regions (Stevens and Wall 2001, Chen et al. 2004, Wallman et al. 2005, Harvey et al. 2008, Reibe et al. 2009, Liu et al. 2011, Boehme et al. 2012, DeBry et al. 2012, Nelson et al. 2012, Sonet et al. 2013). The most comprehensive revision of the genus was published by Aubertin (1933), who recognised 27 species. Since then revisions of the genus and keys for the identification of its species have been produced, but only for specific geographic regions (Hall 1948, James 1971, Rognes 1980, 1991, Smith 1986, Whitworth 2006, 2010). Most species of *Lucilia* are limited to particular continents or islands and very few, such as *Lucilia sericata*, are cosmopolitan. It is difficult to assess relationships and biogeographical patterns when studies are taxonomically geographically fragmented.

At the species level, *Lucilia sericata* and *Lucilia cuprina* have been referred to as sister-species (Ash and Greenberg 1974) because they are very similar morphologically and each is often misidentified as the other. They are now both found in Australia, New Zealand, South Africa, large parts of Asia, Europe and North America (Waterhouse and Paramonov 1950, Rognes 1980, 1994, Norris 1990, Bishop 1991, 1995, Holloway 1991, Fischer 2000, Harvey et al. 2003a, 2003b, 2008, Chen et al. 2004, Heath and Bishop 2006, Park et al. 2009, Liu et al. 2011, Boehme et al. 2012, GilArriortua et al. 2013). They have each received intensive biological investigation, and it would benefit comparative studies if it could be confirmed that they are actually sister species.

Several studies have established that natural hybrids of *Lucilia sericata* and *Lucilia cuprina* exist (Stevens and Wall 1996, Stevens et al. 2002, Wallman et al. 2005, Tourle et al. 2009, DeBry et al. 2010, Williams and Villet 2013). Two other species pairs, *Lucilia coeruleiviridis* Macquart, 1855 and *Lucilia mexicana* Macquart, 1843, and *Lucilia caesar* (Linnaeus, 1758) and *Lucilia illustris* (Meigen, 1826), also show molecular paraphyly (DeBry et al. 2012, Sonet et al. 2012, 2013), possibly due to introgressive hybridisation or incomplete lineage sorting. The frequency and phylogenetic distribution of this phenomenon in the genus is of general interest because of its implications for understanding speciation and diversification in the group.

The aims of this study are therefore to confirm if *Lucilia sericata* and *Lucilia cuprina* are sister-species; to explore if *Lucilia coeruleiviridis* (Macquart, 1855) / *Lucilia mexicana* Macquart, 1843 and *Lucilia caesar* (Linnaeus, 1758) / *Lucilia illustris* (Meigen, 1826) are paraphyletic species; to examine the relationships between the species of *Lucilia* and clarify the taxonomic status of *Phaenicia*; to estimate the relationships of *Dyscritomyia*, *Hemipyrellia*, *Hypopygiopsis* and *Lucilia*; and to assess the geographical and phylogenetic patterns of myiasis-causing behaviour in these flies.

## Materials and methods

### DNA data

Adult *Lucilia* flies were obtained from around the world (Table 1). *Hemipyrellia fernandica* (Macquart, 1855) were obtained from Benin, South Africa and Tanzania, and *Calliphora vicina* Robineau-Desvoidy, 1830 were obtained from France and used as an outgroup (Table 1). Identifications were made by the donors based on morphology and verified using published keys (Aubertin 1931, 1933, Smith 1986, Holloway 1991, Whitworth 2006, 2010). All flies were kept in separate 1.5 ml Eppendorf tubes in 96% ethanol or as dried pinned specimens and deposited with the Durban Natural Science Museum after analysis.

**Table 1.** Specimen locality data for sequences added to GenBank. (Accession numbers starting KF are new sequences from this study).

Species	Specimen	Locality		Accession Number		
				*28S*	*Per*	*COI*
*Calliphora vicina*	CV_FRC_01(F)	Montferrier-Sur-Lez	France	JN792781	KF839531	KF839562
	CV_FRC_02(M)	Montferrier-Sur-Lez	France	KF839506		
*Hemipyrellia fernandica*	H_BEN_01(M)	Contonou	Benin	KF839511	KF839539	KF839567
	H_BEN_02(M)	Contonou	Benin	KF839512	KF839540	KF839568
	H_SA_DBN_01(F)	Durban	South Africa	KF839513	KF839541	KF839569
	H_TAN_01(M)	Mkuraja	Tanzania	KF839514	KF839542	KF839570
	H_TAN_02(M)	Mkuraja	Tanzania	KF839515	KF839543	KF839571
*Lucilia caesar*	Ca_FRC_01(M)	Montferrier-Sur-Lez	France	JN792782	JN792858	KF839556
	Ca_FRC_02(F)	Montferrier-Surz-Lez	France	KF839501	KF839532	KF839557
*Lucilia coeruleiviridis*	Co_CAN_01(M)	Windsor	Canada	KF839502	KF839533	KF839558
	Co_CAN_02(M)	Windsor	Canada	KF839503		KF839559
	Co_USA_03(F)	Putnam Co. Missouri	United States of America	KF839504	KF839534	KF839560
	Co_USA_04(F)	Martinstown, Missouri	United States of America	KF839505		KF839561
*Lucilia cuprina*	C_AUS_01 (M)	Sydney	Australia	KF856254		JN792622
	C_EGT_01 (F)	Alexandria	Egypt	JN792706	JN792784	JN792625
	C_SA_CT_02 (F)	Cape Town	South Africa	JN792713	JN792791	JN792632
	C_SA_DBN_01(F)	Durban	South Africa	JN792724	JN792802	JN792642
	C_THA_02 (F)	Chiang Mai	Thailand	JN792741	JN792819	JN792661
	C_THA_03 (F)	Chiang Mai	Thailand	JN792742	JN792820	JN792662
	C_ZIM_02 (F)	Matobos	Zimbabwe	JN792745	JN792823	JN792667
*Lucilia eximia*	Ex_CSR_01(F)	Santo Domingo	Costa Rica	KF839507	KF839535	KF839563
	Ex_CSR_02(F)	Santo Domingo	Costa Rica	KF839508	KF839536	KF839564
*Lucilia fayeae*	Fa_DOM_01(F)	Calibishie	Dominica	KF839509	KF839537	KF839565
	Fa_DOM_02(F)	Calibishie	Dominica	KF839510	KF839538	KF839566
*Lucilia illustris*	IL_CAN_01(F)	Windsor	Canada	KF839516	KF839544	KF839572
	IL_CAN_02(F)	Windsor	Canada	KF839517	KF839545	KF839573
	IL_JPN_01(F)	Iwate Medical University	Japan	KF839518	KF839546	KF839574
	IL_JPN_02(F)	Iwate Medical University	Japan	KF839519	KF839547	KF839575
	IL_SWZ_01(F)	Lausanne-Suisse	Switzerland	KF839520	KF839548	
	IL_USA_01(F)	Michigan	United States of America	KF839521	KF839549	
	IL_USA_02(F)	Michigan	United States of America	KF839522	KF839550	KF839576
*Lucilia infernalis*	In_BRN_01(F)	Parc National de la Kibira	Burundi	KF839523	KF839551	KF839577
	In_RWN_01(F)	Nyungwe Forest Reserve	Rwanda	JN792780	JN792857	JN813094
*Lucilia mexicana*	Mx_USA_01(F)	New Mexico	United States of America	KF839524	KF839552	KF839578
	Mx_USA_02(F)	New Mexico	United States of America	KF839525		KF839579
*Lucilia papuensis*	Pa_AUS_01	-	Australia	KF839526		
*Lucilia porphyrina*	Po_AUS_01	-	Australia	KF839527	KF839553	
*Lucilia sericata*	S_AUS_01 (M)	Seaford	Australia	JN792746	JN792824	JN792668
	S_FRC_01 (F)	Montferrier-Sur-Lez	France	JN792749	JN792827	JN792671
	S_JPN_01 (F)	Osaka	Japan	JN792754	JN792831	JN792678
	S_NAM_01 (F)	Possession Island	Namibia	JN792758	JN792835	JN792682
	S_SA_CT_07 (F)	Cape Town	South Africa	JN792766	JN792843	JN792690
	S_USA_01 (F)	Michigan	United States of America	JN792778	JN792855	JN792703
*Lucilia silvarum*	Si_GER_01(F)	Kempen	Germany	KF839528		KF839580
*Lucilia thatuna*	Th_USA_01(F)	Del Norte Co. California	United States of America	KF839529	KF839554	KF839581
	Th_USA_02(F)	Del Norte Co. California	United States of America	KF839530	KF839555	KF839582

One hind leg of each fly was used for DNA analysis. DNA was extracted using the Qiagen DNeasy tissue kit (Qiagen, Inc., Valencia, CA) according to the manufacturer’s instructions. Three genes were chosen for sequencing: *28S* rRNA (*28S*), a nuclear gene that has been used in previous studies and would allow comparison with other studies (Stevens et al. 2002, Stevens 2003, Tourle et al. 2009, DeBry et al. 2010, Sonet et al. 2012); *Period (Per)*, a second nuclear gene that is faster-evolving than *28S* to give better phylogenetic resolution; and *Cytochrome oxidase I* (*COI*), the DNA barcoding gene of choice that has been used in previous studies (Stevens et al. 2002, Stevens 2003, Wallman et al. 2005, Wells et al. 2007, Harvey et al. 2008, Liu et al. 2009, Park et al. 2009, Tourle et al. 2009, DeBry et al. 2010, DeBry et al. 2012, Sonet et al. 2012). A region of approximately 650bp in the Domain 1-2 of the *28S* gene was amplified using the primers 5`-CCCCCTGAATTTAAGCATAT-3` and 5`-TTAGACTCCTTGGTCCGTG-3` (Stevens et al. 2002). A region of approximately 600bp of the *COI* gene was amplified using the primers C1-J1709 (5’-ATTGGGGGGTTTGGAAATTG-3`) and C1-N2353 (5’-GCTCGTGTATCAACGTCTATTCC-3`) (Simon et al. 2006). A region of approximately 730bp of the *Per* gene, was amplified using the primers Per5 (5’-GCCTTCAGATACGGTCAAAC-3’) (Warman, pers comm) and Per reverse (5`-CCGAGTGTGGTTTGGAGATT-3`) (designed by the authors). Polymerase chain reaction (PCR) amplification was performed using 1µL of DNA in a 25µL reaction. Amplification times were 94 °C for 5 min denaturation, followed by 36 cycles of 94 °C for 30 seconds, 55 °C for 1 min, 72 °C for 30 seconds and a final extension period at 72 °C for 7 min. PCR products were confirmed by gel electrophoresis stained in ethidium bromide. PCR products were then sequenced using an ABI 3730l Genetic Analyzer (Applied Biosystems) and the primers used in amplification.

Additional DNA sequences of *28S*, *Per* and *COI* were obtained from GenBank (www.ncbi.nlm.nih.gov) (Table 2). Additional *COI* barcode sequences were downloaded from the Barcode of Life Database (BOLD) website for all available *Lucilia*, *Hemipyrellia* and *Hypopygiopsis* species and for *Paralucilia paraensis* (Mello, 1972) and *Chrysomya chloropyga* (Wiedemann, 1818) which were included as additional outgroups. Duplicate sequences from the same studies were removed and a total of 207 sequences were included in the analysis. The sequences were aligned and edited using the BioEdit v7.0.9 software (Hall 1999).

**Table 2.** GenBank sequences included in this study.

Species	Locality		Accession Number		
			*28S*	*Per*	*COI*
*Calliphora vicina*	Bristol	UK	AJ300131		AJ417702
*Dyscritomyia fasciata*	-	Hawaii			AY074902
*Dyscritomyia lucilioides*	-	Hawaii			AY074903
*Dyscritomyia robusta*	-	Hawaii			AY074898
*Hemipyrellia ligurriens*	-	China			DQ345092
*Hemipyrellia ligurriens*	-	Taiwan			AY097334
*Hemipyrellia ligurriens*	-	Taiwan			DQ453493
*Hemipyrellia pulchra*	-	China			DQ345091
*Lucilia adiosoemartoi*	-	Indonesia			AY074901
*Lucilia ampullacea*	Langford	UK	AJ300137		
*Lucilia ampullacea*	Bristol	UK			DQ453487
*Lucilia ampullacea*	-	Korea			EU925394
*Lucilia bazini*	-	Taiwan			AY346450
*Lucilia bazini*	-	China			DQ345082
*Lucilia caesar*	Langford	UK	AJ300138		AY417703
*Lucilia caesar*	Bristol	UK			DQ453488
*Lucilia caesar*	-	Korea			EU880196
*Lucilia cluvia*	New Orleans	USA	AJ551440		DQ453490
*Lucilia cluvia*	Volusia Co. Florida	USA			JQ942371
*Lucilia coeruleiviridis*	New York	USA			FJ650558
*Lucilia cuprina*	-	China			DQ345087
*Lucilia cuprina*	Honolulu	Hawaii			AJ417704
*Lucilia cuprina*	Oahu	Hawaii			DQ453496
*Lucilia cuprina*	-	Taiwan			AY097335
*Lucilia cuprina*	-	Thailand			EU418577
*Lucilia cuprina*	Tororo	Uganda			AJ417711
*Lucilia cuprina*	Townsville	Australia	AJ417709		AJ417710
*Lucilia cuprina*	Waianae	Hawaii			AJ417705
*Lucilia cuprina*	Wallaceville	New Zealand		Y19108.1	
*Lucilia cuprina*	Noordhoek	South Africa	EU626549		
*Lucilia cuprina*	Cincinnati	USA	FJ650542		
*Lucilia eximia*	-	Brazil			DQ453491
*Lucilia hainanensis*	-	Taiwan			AY346451
*Lucilia hainanensis*	-	China			DQ345084
*Lucilia illustris*	Langford	UK	AJ300136		AJ551445
*Lucilia illustris*	-	Korea			EU880204
*Lucilia illustris*	-	China			DQ345090
*Lucilia illustris*	-	India			DQ200168
*Lucilia mexicana*	San Francisco	USA	AJ551441		DQ453492
*Lucilia mexicana*	California	USA			FJ650563
*Lucilia mexicana*	California	USA			FJ650562
*Lucilia papuensis*	-	China			DQ345085
*Lucilia porphyrina*	-	Taiwan			AY097336
*Lucilia porphyrina*	-	Japan			AY074900
*Lucilia porphyrina*	-	China			DQ345089
*Lucilia richardsi*	Usk	-	AJ551142		
*Lucilia sericata*	Perth	Australia			AB112833
*Lucilia sericata*	Nerja	Spain			AJ417716
*Lucilia sericata*	Kingsbury	UK			AJ417713
*Lucilia sericata*	Hilerod	Denmark	AJ300140		EF531193
*Lucilia sericata*	Harare	Zimbabwe			AJ417717
*Lucilia sericata*	-	China			DQ345086
*Lucilia sericata*	Langford	UK	AJ300139		
*Lucilia sericata*	Los Angeles	USA	AJ300141		
*Lucilia silvarum*	Durham	UK	AJ551443		
*Lucilia silvarum*	-	USA			FJ650564
*Lucilia silvarum*	Linn Co., OR	USA			JQ942455
*Lucilia taiyuanensis*	-	China			DQ345088
*Lucilia thatuna*	San Francisco	USA	AJ551444		DQ453489
*Lucilia thatuna*	Del Norte Co., California	USA			JQ942464

### Morphological data

The states of the 14 morphological characters defined by Stevens and Wall (1996) were obtained from Aubertin (1931, 1933), Stevens and Wall (1996) and Whitworth (2010) for all of the *Lucilia* and *Hemipyrellia* species for which sequences were available (Table 3). Museum specimens were inspected where possible to complete the character state matrix. *Calliphora vicina* was included as an outgroup.

**Table 3.** Binary coding of 14 morphological characters for the genera *Lucilia* and *Hemipyrellia*. 1 – Colour of the basicostal scale (0 = black/brown, 1 = white/cream); 2 – Number of postsutural acrostichal bristles (0 = two pairs, 1 = three pairs); 3 – Eye separation in the male (0 = distance of greater than the width of the third antennal segment, 1 = less than the width of the third antennal segment); 4 – Number of anterio-dorsal bristles on the mid tibia (0 = one, 1 = two); 5 – Colour of the palpi (0 = yellow/orange, 1 = black/brown); 6 – Subcostal sclerite (0 = bristles absent, 1 = bristles present); 7 – Colour of the squamae (0 = uniform white/cream, 1 = partially or totally brown); 8 – Wings (00 = hyaline, 01 = lightly infuscated, 11 = heavily infuscated); 9 – Eye separation in the female (0 = distance of greater than one quarter of the width of the head, 1 = less than one quarter of the width of the head); 10 – Colour of antennae (0 = uniformly dark, 1 = non-uniform); 11 – Male hypopygium (00 = inconspicuous, 01 = conspicuous, 11 = highly conspicuous); 12 – Colour of abdomen and thorax (0 = predominantly brassy green/green, 1 = predominantly purple/blue/black); 13 – Colour of the legs (00 = dark brown, 01 = brown/black, 11 = black); 14 – Lower squamal lobe (0 = setae absent, 1 = setae present). (Stevens and Wall 1996).

Species	Character number													
	1	2	3	4	5	6	7	8	9	10	11	12	13	14
*Calliphora vicina*	1	1	1	1	0	0	1	00	0	0	11	1	01	1
*Hemipyrellia fernandica*	0	0	0	0	1	1	0	00	1	0	00	0	11	0
*Hemipyrellia ligurriens*	0	0	0	0	0	1	0	00	1	1	01	0	11	0
*Hemipyrellia pulchra*	0	0	0	1	0	?	0	00	0	1	00	0	11	0
*Lucilia ampullacea*	0	0	1	0	0	1	0	00	0	0	00	0	01	0
*Lucilia bufonivora*	0	0	0	0	1	0	0	00	0	0	01	0	11	0
*Lucilia coeruleiviridis*	1	0	1	0	0	0	0	00	1	1	00	0	00	0
*Lucilia caesar*	0	0	1	0	0	1	0	00	0	0	11	0	01	0
*Lucilia cluvia*	1	0	0	0	0	0	0	00	1	0	00	0	00	0
*Lucilia cuprina*	1	1	0	0	0	0	0	00	0	0	01	0	11	0
*Lucilia eximia*	0	0	1	0	0	0	1	00	0	1	00	0	00	0
*Lucilia fayeae*	0	0	1	0	0	0	1	01	0	0	00	1	00	0
*Lucilia illustris*	0	0	1	0	0	1	0	00	0	0	01	0	11	0
*Lucilia infernalis*	0	1	1	0	0	1	1	11	0	1	00	1	01	0
*Lucilia mexicana*	0	0	1	0	0	0	1	01	0	1	00	0	11	0
*Lucilia papuensis*	0	0	1	1	0	1	1	01	0	1	00	0	11	0
*Lucilia porphyrina*	0	0	1	0	0	1	1	01	1	0	00	1	00	0
*Lucilia richardsi*	1	1	0	1	1	0	0	00	0	0	00	0	11	0
*Lucilia sericata*	1	1	0	0	0	0	0	00	0	0	00	0	11	0
*Lucilia silvarum*	0	1	0	0	1	0	0	00	0	0	01	0	11	0
*Lucilia thatuna*	1	1	1	0	0	0	0	00	1	0	00	0	11	0

### Phylogenetic analysis

Separate Bayesian inference analyses were performed on each gene in MrBayes (Huelsenbeck and Ronquist 2001) using the best-fitting nucleotide substitution model (GTR+G in all cases) from jModelTest (Posada 2008). One cold and three hot chains were run for 5 000 000 generations, sampling every 1 000 generations with burn-in of 1 000 samples (20%). Incongruence length difference (ILD) tests (Farris et al. 1994) were run in PAUP*4b10 (Swofford 2003) to quantify the differences in topology between trees for *28S*, *COI* and *Per*. Analyses were then conducted on two combined data sets (nuclear *28S* and *Per*; and total *28S*, *Per* and *COI*), each partitioned by gene, with the parameters as above.

A network analysis for the *COI* data was created using the NeighborNet algorithm in SplitsTree4 (Huson and Bryant 2008) and the uncorrected P-distance method.

The *COI* barcode sequences (~700 bp long, between base numbers 1490 and 2198) retrieved from on-line databases were aligned along with our new sequences (~640 bp long, between base numbers 1709 and 2353) for a region approximately 800 bp long in which every sequence overlapped the others by at least 490 bp. Bayesian inference analysis was performed in MrBayes (Huelsenbeck and Ronquist 2001) using the best-fitting nucleotide substitution model (GTR+G) from jModelTest (Posada 2008).

Maximum parsimony analysis of the morphological data (Table 3) using Fitch parsimony was performed in Paup*4b10 (Swofford 2003). Statistical support for nodes was assessed by bootstrapping with 100 replicates retaining a maximum of 10 000 trees. Strict consensus and 50% majority rule trees were produced from the analysis.

The zoogeographic distributions of species in the Luciliinae (Table 4) were mapped onto the trees.

**Table 4.** Zoogeographic distribution of species of Luciliinae included in this study. Symbols in brackets represent anthropogenic introductions.

Species	Region						
	Hawaii	Afrotropical	Australasian	Oriental	Palaearctic	Neararctic	Neotropical
*Dysctritomyia* spp.	X						
*Hypopygiopsis* spp.			X	X			
*Hemipyrellia* spp.		X	X	X			
*Hemipyrellia fernandica*		X					
*Lucilia infernalis*		X					
*Lucilia cuprina*		X	X	X	(X)	X	
*Lucilia sericata*		(X)	(X)	X	X	X	(X)
*Lucilia silvarum*					X	X	
*Lucilia thatuna*						X	
*Lucilia adiosoemartoi*				X			
*Lucilia bazini*				X			
*Lucilia hainanensis*				X			
*Lucilia taiyuanensis*				X			
*Lucilia papuensis*			X	X			
*Lucilia porphyrina*			X	X	X		
*Lucilia ampullacea*				X	X		
*Lucilia caesar*				X	X		
*Lucilia illustris*				X	X	X	
*Lucilia cluvia*					X	X	
*Lucilia coeruleiviridis*						X	
*Lucilia mexicana*						X	
*Lucilia fayeae*							X
*Lucilia eximia*							X

## Results

### Molecular data

Sequencing of the *28S*, *Per* and *COI* genes resulted in 1932 bp being aligned – 656 bp for *28S*, 700 bp for *Per* and 576 bp for *COI*. A total of 46 specimens were sequenced for *28S*, 41 specimens for *Per* and 39 specimens for *COI*. These sequences were submitted to GenBank (Table 1).

The ILD test for *28S* and *Per* showed these two genes to be highly congruent (p = 1.00) and the datasets were therefore concatenated for the analyses. The ILD test for *28S*, *Per* and *COI* showed the combination of these genes to be incongruent (p = 0.03). Despite the incongruence between the nuclear (*28S* and *Per*) and mitochondrial (*COI*) data, these data sets were also concatenated and an analysis run on the total molecular evidence.

**Figure 1.**
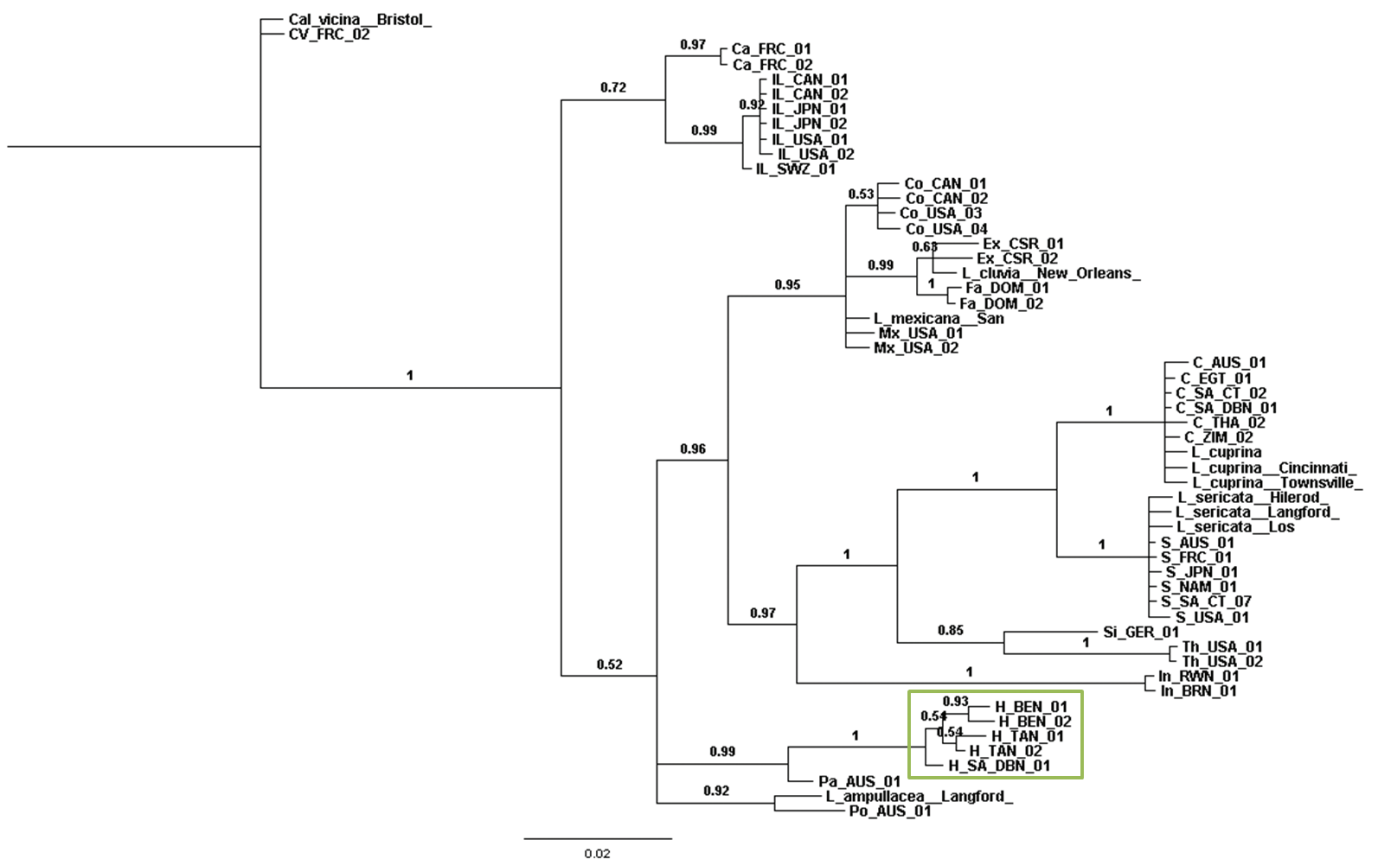
Bayesian inference tree constructed from concatenated nuclear genes *28S* + *Per*. Posterior probabilities are indicated on nodes. Green box = *Hemipyrellia fernandica*. C = *Lucilia cuprina*, Ca = *Lucilia caesar*, Co = *Lucilia coeruleiviridis*, CV = *Calliphora vicina*, Ex = *Lucilia eximia*, Fa = *Lucilia fayeae*, H = *Hemipyrellia fernandica*, IL = *Lucilia illustris*, In = *Lucilia infernalis*, Mx = *Lucilia mexicana*, Pa = *Lucilia papuensis*, Po = *Lucilia porphyrina*, S = *Lucilia sericata*, Si = *Lucilia silvarum*, Th = *Lucilia thatuna*, AUS = Australia, BRN = Burundi, CAN = Canada, CSR = Costa Rica, DOM = Dominican Republic, FRC = France, GER = Germany, JPN = Japan, NAM = Namibia, EGT = Egypt, RWN = Rwanda, SWZ = Switzerland, SA = South Africa, TAN = Tanzania, THA = Thailand, USA = United States of America, ZIM = Zimbabwe. DBN = Durban, CT = Cape Town.

The Bayesian inference tree (Fig. 1) for the nuclear genes (*28S* and *Per*) clearly showed that *Lucilia sericata* and *Lucilia cuprina* are sister clades with 100% support. *Lucilia thatuna* Shannon, 1926 and *Lucilia silvarum* Meigen, 1826 form a sister clade to the *Lucilia sericata + Lucilia cuprina* clade. The specimens of *Hemipyrellia fernandica* all grouped together and were sister to *Lucilia papuensis* Macquart, 1843. The *Hemipyrellia* clade sat within the *Lucilia* clade (Fig. 1).

**Figure 2.**
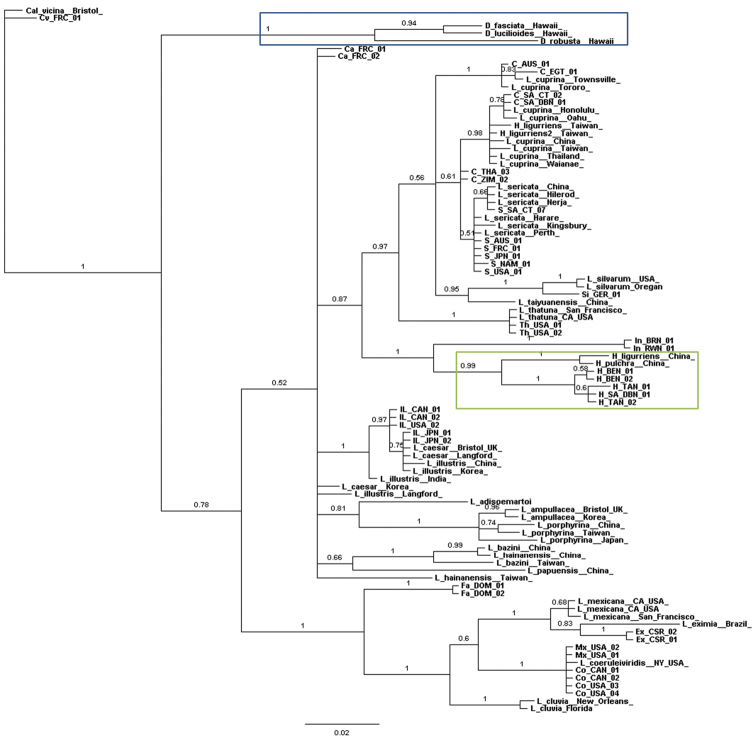
Bayesian inference tree constructed from mitochondrial gene *COI*. Posterior probabilities indicated on nodes. Green box = *Hemipyrellia* sp. Blue box = *Dysctritomyia* sp. C = *Lucilia cuprina*, Ca = *Lucilia caesar*, Co = *Lucilia coeruleiviridis*, CV = *Calliphora vicina*, Ex = *Lucilia eximia*, Fa = *Lucilia fayeae*, H = *Hemipyrellia fernandica* IL = *Lucilia illustris*, In = *Lucilia infernalis*, Mx = *Lucilia mexicana*, S = *Lucilia sericata*, Si = *Lucilia silvarum*, Th = *Lucilia thatuna*, AUS = Australia, BRN = Burundi, CAN = Canada, CSR = Costa Rica, DOM = Dominican Republic, FRC = France, GER = Germany, JPN = Japan, NAM = Namibia, EGT = Egypt, RWN = Rwanda, SWZ = Switzerland, SA = South Africa, TAN = Tanzania, THA = Thailand, USA = United States of America, ZIM = Zimbabwe. DBN = Durban, CT = Cape Town.

In the Bayesian inference tree for the mitochondrial gene (*COI*) (Fig. 2), *Lucilia cuprina* was paraphyletic with respect to *Lucilia sericata*. The *Lucilia cuprina* + *Lucilia sericata* clade was poorly resolved with respect to the *Lucilia silvarum* + *Lucilia taiyuanensis* Chu, 1975 clade. The *Hemipyrellia fernandica* sequences grouped with those of *Hemipyrellia ligurriens* and *Hemipyrellia pulchra* from GenBank and this clade was sister to *Lucilia infernalis* Villeneuve, 1914. This *Hemipyrellia* + *Lucilia infernalis* clade sat within the *Lucilia* clade on the tree. Two specimens from Taiwan assigned to *Hemipyrellia ligurriens* grouped with the *Lucilia cuprina* specimens. The three *Dyscritomyia* sequences included in the analysis grouped together monophyletically outside *Lucilia*.

**Figure 3.**
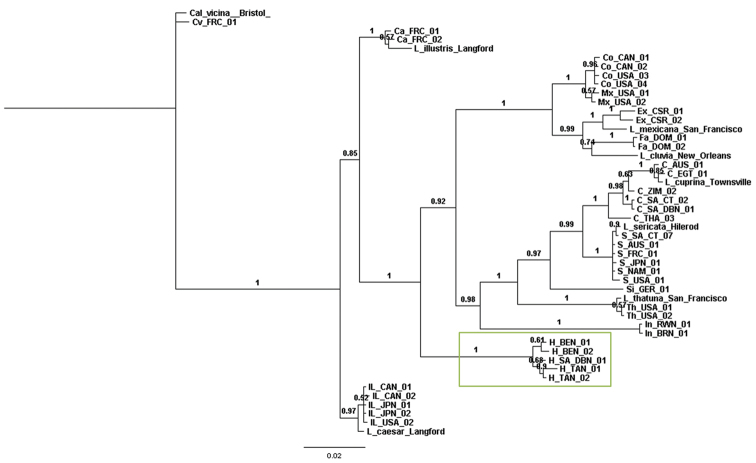
Bayesian inference tree constructed from the concatenated nuclear (*28S* & *Per*) and mitochondrial (*COI*) genes. Posterior probabilities indicated on nodes. Green box = *Hemipyrellia fernandica*. C = *Lucilia cuprina*, Ca = *Lucilia caesar*, Co = *Lucilia coeruleiviridis*, CV = *Calliphora vicina*, Ex = *Lucilia eximia*, Fa = *Lucilia fayeae*, H = *Hemipyrellia fernandica* IL = *Lucilia illustris*, In = *Lucilia infernalis*, Mx = *Lucilia mexicana*, S = *Lucilia sericata*, Si = *Lucilia silvarum*, Th = *Lucilia thatuna*, AUS = Australia, BRN = Burundi, CAN = Canada, CSR = Costa Rica, DOM = Dominican Republic, FRC = France, GER = Germany, JPN = Japan, NAM = Namibia, EGT = Egypt, RWN = Rwanda, SWZ = Switzerland, SA = South Africa, TAN = Tanzania, THA = Thailand, USA = United States of America, ZIM = Zimbabwe. DBN = Durban, CT = Cape Town.

The Bayesian inference tree for the incongruent concatenated total evidence molecular dataset (*28S*, *Per* and *COI*) (Fig. 3) showed *Lucilia sericata* and *Lucilia cuprina* to be sister clades with strong support. The *Hemipyrellia fernandica* sequences sat within *Lucilia*, and the rest of the tree was topologically similar to the gene trees.

**Figure 4.**
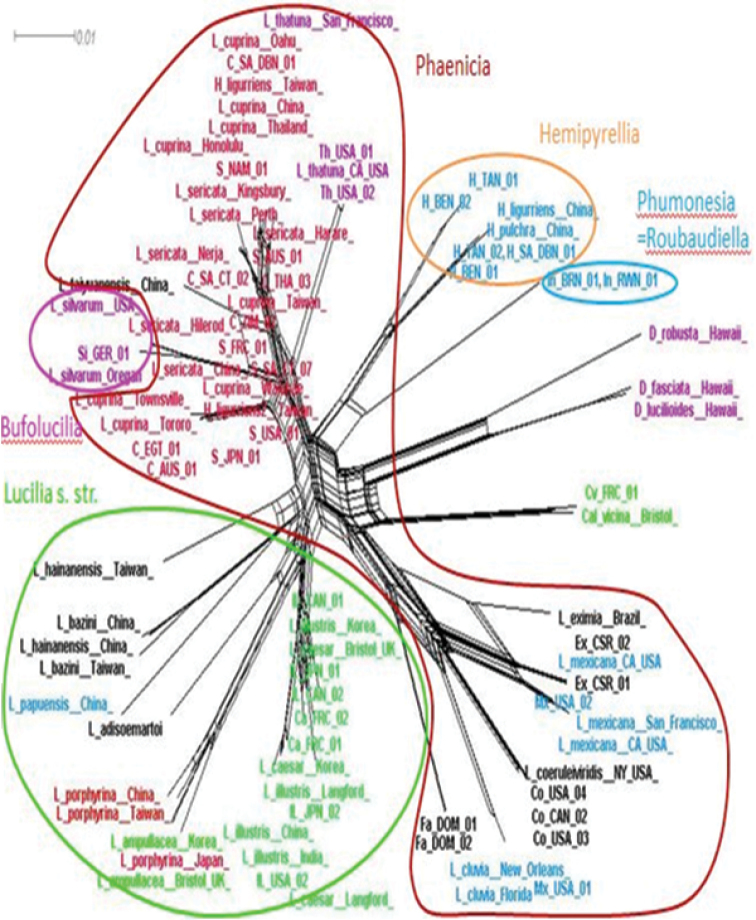
NeighborNet network diagram constructed from *COI* data showing parasitic behaviour (coloured text) and previous sub-generic status of *Lucilia* according to Hall (1948) (ellipses). Text colours: Red = primary facultative parasite, green = secondary facultative parasite, purple = parasite (unknown if primary or secondary), blue = saprophage, black = unknown parasitic behaviour. C = *cuprina*, Ca = *caesar*, Co = *coeruleiviridis*, CV = *Calliphora vicina*, Ex = *eximia*, Fa = *fayeae*, H = *Hemipyrellia fernandica*, IL = *illustris*, In = *infernalis*, Mx = *mexicana*, S = *sericata*, Si = *silvarum*, Th = *thatuna*, AUS = Australia, BRN = Burundi, CAN = Canada, CSR = Costa Rica, DOM = Dominica, FRC = France, GER = Germany, JPN = Japan, NAM = Namibia, EGT = Egypt, RWN = Rwanda, SWZ = Switzerland, SA = South Africa, TAN = Tanzania, THA = Thailand, USA = United States of America, ZIM = Zimbabwe. DBN = Durban, CT = Cape Town.

The NeighborNet analysis (Fig. 4) clearly showed seven distinct major splits. The New World species (*Lucilia coeruleiviridis*, *Lucilia cluvia* Walker, 1849, *Lucilia eximia* Wiedemann, 1819, *Lucilia mexicana* and *Lucilia fayeae* Whitworth, 2010) grouped together; *Lucilia caesar*, *Lucilia illustris*, *Lucilia porphyrina* Walker, 1856, *Lucilia ampullacea* Villeneuve, 1922, *Lucilia adiosoemartoi*, *Lucilia papuensis* Macquart, 1843, *Lucilia bazini* Séguy, 1934 and *Lucilia hainanensis* Fan, 1965 formed a group; *Lucilia infernalis* was isolated, as was *Hemipyrellia fernandica*; the bulk of the *Lucilia* species that are primary facultative parasites (*Lucilia sericata*, *Lucilia cuprina*, *Lucilia silvarum* and *Lucilia thatuna*) grouped together; and *Calliphora vicina* and the *Dyscritomyia* species as the outgroups formed separate but neighbouring splits.

**Figure 5.**
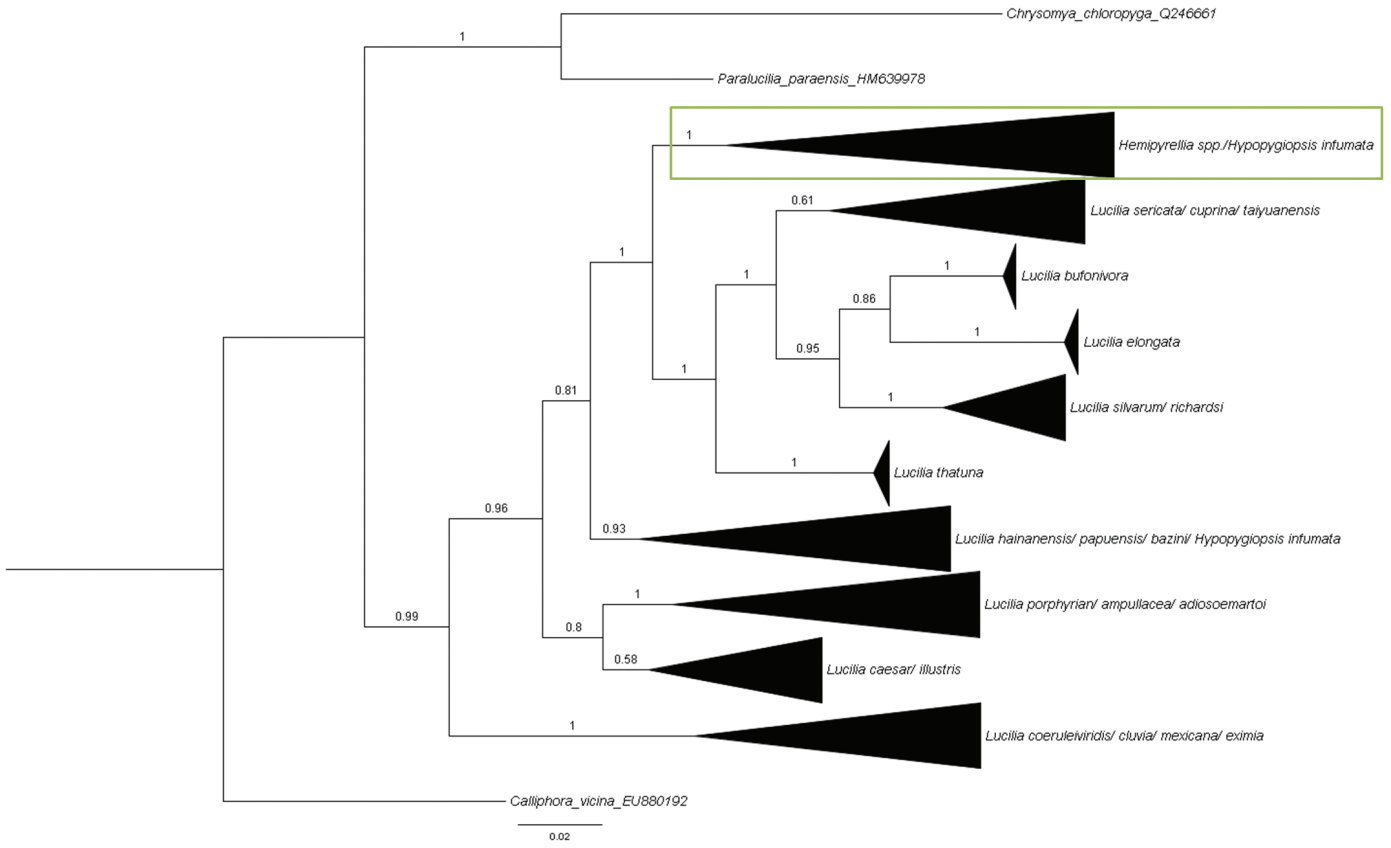
Bayesian inference tree constructed using *COI* barcode sequences. Posterior probabilities indicated on nodes. Support within the collapsed nodes is variable. Green box = *Hemipyrellia* sp.

Bayesian inference analysis of the *COI* barcode data set generated a tree (Fig. 5) with very strong posterior probabilities for most clades except for the *Lucilia sericata + Lucilia cuprina* + *Lucilia taiyuanensis* (p = 0.61) and *Lucilia caesar* + *Lucilia illustris* (p = 0.58) clades. The *Hemipyrellia* species all formed a distinct clade within *Lucilia* with 100% support. One of the *Hypopygiopsis infumata* (Bigot, 1877) sequences forms a clade with *Lucilia hainanensis* + *Lucilia papuensis* + *Lucilia bazini* and the other sequence groups with the *Hemipyrellia* sequences. *Paralucilia paraensis* sat outside *Lucilia* with *Chrysomya chloropyga*, confirming its classification as a chrysomyine.

### Morphological data

**Figure 6.**
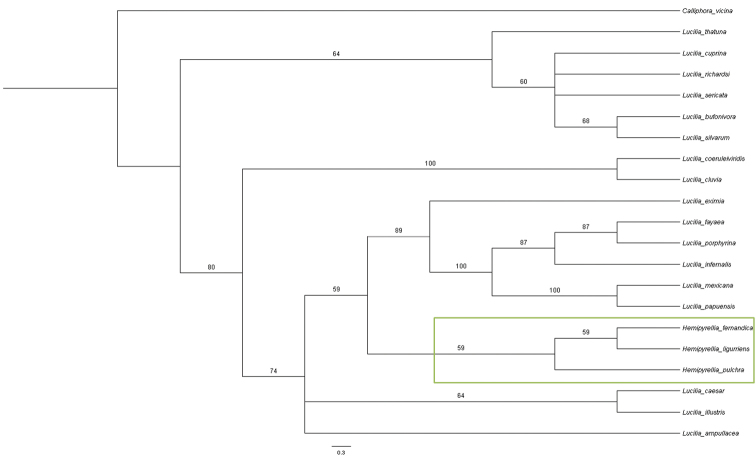
Majority rule consensus tree for 21 species of *Lucilia* and *Hemipyrellia* constructed from morphological characters listed in Table 3. Green box = *Hemipyrellia* sp.

The strict consensus parsimony tree for the morphological characters was largely uninformative, forming only two clades, with the majority of the species being unresolved (tree not shown). The 50% majority rule consensus tree (Fig. 6) grouped *Lucilia sericata*, *Lucilia cuprina*, *Lucilia silvarum*, *Lucilia bufonivora* and *Lucilia thatuna* together. *Lucilia coeruleiviridis* and *Lucilia cluvia* grouped together in all of the trees. The *Hemipyrellia* species formed a clade within *Lucilia*, and *Lucilia caesar* and *Lucilia illustris* grouped together.

## Discussion

The majority rule consensus tree of the morphological characters (Fig. 6) was largely incongruent with the molecular phylogenetic trees (Figs 1–3, 5). The only clade that was congruent contains *Lucilia sericata* + *Lucilia cuprina* + *Lucilia richardsi* + *Lucilia silvarum* + *Lucilia bufonivora* + *Lucilia thatuna*. In the *COI* Bayesian inference tree this clade included *Lucilia elongata* too (Fig. 5). This is partly due to disparities in taxon sampling and possibly partly a result of the limited character set available for the morphological parsimony analysis. It is ideal to have at least three times more characters than species in this type of analysis (Stevens and Wall 1996), whereas the matrix has 21 species and 17 character states, which limits the conclusions about general trends that can be drawn from these morphological data. This discussion will therefore focus on the results of the molecular analyses.

### Relationship of *Lucilia sericata* and *Lucilia cuprina*

Although only about half of the *Lucilia* species listed as valid by Aubertin (1933) were included in this study, these results strongly suggest that *Lucilia sericata* and *Lucilia cuprina* are indeed sister species. All of the Bayesian inference analyses (Figs 1–3) indicated this with strong support from the nuclear genes (*28S* and *Per*) and total evidence (*28S* + *Per* + *COI*) trees and weaker support from the *COI* gene alone. *Lucilia cuprina* was paraphyletic (Fig. 2) with respect to *Lucilia sericata* in the mitochondrial gene (*COI*) tree, as shown previously (using the same sequences but weaker auxiliary taxon sampling) to be the result of introgressive hybridisation between these two species (Williams and Villet 2013). In another study (McDonagh and Stevens 2011), the nuclear gene elongation factor-1 alpha (*EF-1α*) did not recover *Lucilia sericata* and *Lucilia cuprina* as sister-species, but the clade containing *Lucilia sericata* was poorly resolved and thus the conclusion was weakly supported, but the *28S* and *COI* gene trees both recovered *Lucilia sericata* and *Lucilia cuprina* as sister species with strong support (McDonagh and Stevens 2011).

### Molecular identification of *Lucilia* species

It has already been established that *Lucilia sericata* and *Lucilia cuprina* show a case of ancient introgression, and that they still interbreed (Williams and Villet 2013). This is a widely acknowledged problem for identification using partial *COI* sequences alone (Rubinoff et al. 2006, Nelson et al. 2007, Roe and Sperling 2007, Williams et al. 2008, Tantawi et al. 2010, Williams and Villet 2013). Other problematic species pairs occur in the genus (DeBry et al. 2012, Sonet et al. 2012), and it is important to recognise the cause(s) and to document genes that are more useful for identification in these contexts.

In the Bayesian inference trees based on mitochondrial (*COI*) (Fig. 2) and total evidence (*28S*, *Per* and *COI*) (Fig. 3), *Lucilia mexicana* was paraphyletic with respect to *Lucilia coeruleiviridis*. This has been observed in the continental United States of America (DeBry et al. 2012), where these two species were found to share a mitochondrial haplotype. The *Lucilia mexicana* specimens with this *Lucilia coeruleiviridis* haplotype appear to be limited to a geographic area including Texas and New Mexico (DeBry et al. 2012). This study independently confirms this pattern, since our new sequences of *Lucilia mexicana* from New Mexico grouped with *Lucilia coeruleiviridis*, and the GenBank specimens of *Lucilia mexicana* from California formed a distinct clade (Figs 2–3). This suggests introgression between *Lucilia mexicana* and *Lucilia coeruleiviridis*. The nuclear genes separated *Lucilia coeruleiviridis* and *Lucilia mexicana*, although *Lucilia mexicana* was not resolved in this analysis (Fig. 1). In the Bayesian inference tree based on the *Per* gene alone (tree not shown), these two species are recovered as sister clades with 100% support, which suggests that nuclear genes will separate these two species as they do for *Lucilia sericata* and *Lucilia cuprina* (Williams and Villet 2013).

*Lucilia caesar* and *Lucilia illustris* also share haplotypes (Sonet et al. 2012). In the *COI* tree (Fig. 2), *Lucilia caesar* specimens from France and Korea and one specimen of *Lucilia illustris* from the UK were not resolved, but the remainder of the *Lucilia caesar* and *Lucilia illustris* specimens formed a mixed clade with 100% support. These two species can therefore not be unambiguously identified using only *COI*. The nuclear genes in this study (Fig. 1) separated these two species but used only two specimens of *Lucilia caesar* from France and seven specimens of *Lucilia illustris* from Japan, Switzerland, Canada and the United States of America. Including specimens from other countries may give a different result as was seen in a previous study (Sonet et al. 2012) where *Lucilia caesar* and *Lucilia illustris* could not be reliably identified using either mitochondrial or nuclear genes as the intraspecific and interspecific genetic distances were very low. This might result from hybridisation or incomplete lineage sorting (Sonet et al. 2012).

These three species pairs highlight the need for using more than one gene to identify species, as has been suggested in previous studies (Rubinoff et al. 2006, Nelson et al. 2007, Roe and Sperling 2007, Williams et al. 2008, Tantawi et al. 2010, Williams and Villet 2013). It also highlights a problem in using *COI* as a universal ‘barcoding’ gene (Rubinoff et al. 2006, Roe and Sperling 2007, Whitworth et al. 2007, Sonet et al. 2012, van Nieukerken et al. 2012, Jordaens et al. 2013), especially in a forensic context. While cases of ancient introgression remain genetically identifiable (DeBry et al. 2012, Williams and Villet 2013), cases of incomplete lineage sorting may be intractable, and morphological identification may be the best solution, especially if the identifications need to go to court.

### Diversification of Luciliinae

The Luciliinae showed two strong patterns underlying their diversification: biogeographical radiation and the diversification of parasitism.

The analyses (summarised in Fig. 4) showed geographically distinct clusters of species from the New World (*Lucilia eximia* + *Lucilia mexicana* + *Lucilia coeruleiviridis* + *Lucilia cluvia* + *Lucilia fayeae*), the Oriental region (*Lucilia hainanensis* + *Lucilia bazini* + *Lucilia papuensis* + *Lucilia adiosoemartoi* Kurahashi, 1988), and Eurasia (*Lucilia porphyrina* + *Lucilia ampullacea*). *Hemipyrellia* formed a monophyletic Old World lineage (Aubertin 1931). *Lucilia infernalis* is found only in Africa (Aubertin 1933) and the sequences from Rwanda and Burundi formed a separate group. One component of phylogenetic diversification within *Lucilia* is therefore certainly biogeographical.

*Lucilia sericata*, *Lucilia cuprina*, *Lucilia thatuna* and *Lucilia silvarum* form a clade of facultatively parasitic species, with *Lucilia sericata* and *Lucilia cuprina* being primary facultative parasites. This group is geographically diverse, with only *Lucilia thatuna* being restricted to one region, the United States of America. Likewise, *Lucilia caesar* and *Lucilia illustris* form a clade that represents secondary facultative parasites. *Lucilia illustris* is Holarctic, while *Lucilia caesar* is restricted to the Palaearctic (DeBry et al. 2012). *Dyscritomyia* is endemic to the Hawaiian Islands (Wells et al. 2002) and phylogenetically coherent. Its members are attracted to carrion and are suspected of breeding in carrion and parasitizing snails (Hardy 1981).

Many *Lucilia* species are myiasis-causing (Zumpt 1965), with *Lucilia cuprina* being the most recognised and often referred to as the sheep-strike blowfly (Hepburn 1943, Ullyett 1945, Vogt and Woodburn 1979, Heath and Bishop 2006). Other species of *Lucilia* known to be facultative parasites include *Lucilia sericata*, *Lucilia silvarum*, *Lucilia thatuna*, *Lucilia richardsi*, *Lucilia porphyrina*, *Lucilia illustris*, *Lucilia caesar*, and *Lucilia ampullacea*; the only obligately parasitic species in the genus are *Lucilia bufonivora* and possibly *Lucilia elongata* (Aubertin 1933, Hall 1948, Zumpt 1965, Rognes 1991, McDonagh and Stevens 2011). There are also saprophagous species within *Lucilia*, including *Lucilia mexicana*, *Lucilia cluvia*, *Lucilia papuensis* and *Lucilia infernalis* (Hall 1948, Zumpt 1965). None of these different parasitic behaviours are limited to any particular geographical area (Fig. 4). This implies that diversification of breeding behaviours has also been a component of phylogenetic diversification within *Lucilia*, independent of biogeography.

## Taxonomy of Luciliinae

***Lucilia*** Robineau-Desvoidy, 1830 (type species: *Lucilia caesar* (Linnaeus, 1758) has a complex nomenclatural history that is integrally related to its biogeographical and dietary radiation. Several authors including Bigot, van der Wulp, Brauer and Bergenstamm, Girschner, Hough, Kramer, Shannon and Malloch (Aubertin 1933) contributed to the ultimate development of this genus. Early studies of the European *Lucilia* were conducted by Stein (1924), Richards (1926), Collin (1926) and Séguy (1928) and Shannon published on the North and South Amercican *Lucilia* (1926) (Aubertin 1933). Aubertin (1933) published the most comprehensive review of the genus and recognised 27 species. This genus is widely spread across with world. The adults of this genus feed on nectar, carrion and decomposing material and the females are oviparous (Aubertin 1933). The larvae of this genus develop on decomposing animal material. Several species have developed specialised parasitic behaviour such as *Lucilia cuprina*, which lays its eggs on living sheep and the larvae feed on the live animals, causing myiasis. *Lucilia bufonivora* is a parasite of toads.

***Phaenicia*** Robineau-Desvoidy, 1863 (type species: *Phaenicia concinna* Robineau-Desvoidy, 1863 = *Musca sericata* Meigen, 1826) has a history of varied usage. Hall (1948) divided *Lucilia* into several separate genera including *Bufolucilia*, *Phaenicia* and *Lucilia* *sensu stricto.* Hall’s (1948) separation of species into the genera *Phaenicia* and *Lucilia* was primarily based on the presence or absence of bristles on the subcostal sclerite and the character of the ocellar triangle. In contrast, Malloch (1926) used the yellow colour of the basicostal scale and the presence of three postsutural acrostichal bristles to define his concept of *Phaenicia*. The use of *Phaenicia* has persisted in North American literature (Stevens and Wall 1996, Byrd and Castner 2010), but is not generally used in other parts of the world as it is seen as a junior synonym of *Lucilia* (Zumpt 1965).

In the network analysis (Fig. 4), the species that would be assigned to *Phaenicia* based on Hall’s (1948) criteria can clearly be seen to be part of two distant clades. These species occur in both the Old and New Worlds, showing vast geographic ranges. The group includes species that are primary facultative parasites and species that are saprophages. Hall’s (1948) usage of *Lucilia* s.str. refers only to *Lucilia illustris* (and *Lucilia caesar* for clarity between the two) as he focused only on Nearctic blowflies. The remaining species that would fall into this clade based on his diagnostic criteria grouped with *Lucilia caesar* and *Lucilia illustris* in our analyses (Fig. 4), and includes species that are primary and secondary facultative parasites as well as species that are saprophagous.

***Bufolucilia*** Townsend, 1919 (type species: *Lucilia bufonivora*) includes the species *bufonivora*, *silvarum* and (by monophyly) *elongata*, which are found in Europe and North America. *Bufolucilia* forms a part of the clade that includes most of the facultatively parasitic *Lucilia* species (Fig. 4). There is no obvious reason to separate *Lucilia* into (sub)genera based on the parasitic behaviour of the species because primary and secondary facultatively parasitic and saprophagous species are spread throughout the genus (Fig. 4). Recognising *Bufolucilia* also makes *Phaenicia* paraphyletic (Fig. 4).

***Phumonesia*** Villeneuve, 1914 and ***Roubaudiella*** Séguy, 1925 (type species: *Phumonesia infernalis* Villeneuve, 1914 = *Roubaudiella caerulea* Robineau-Desvoidy, 1863) are monotypic genera founded on the same species, and therefore objective synonyms. The only species shows affinities with *Hemipyrellia* in some analyses (Fig. 2, 4), and is always embedded inside *Lucilia*, leaving no reason to recognise a separate genus.

Similarly, *Francilia* Shannon, 1924, and *Acrophagella* Ringdahl, 1942, are objective synonyms because they are based on the same species. Several other genus-group taxa have been erected within the Luciliinae, including *Caesariceps* Rodendorf, 1926, *Dasylucilia* Rodendorf, 1926, *Luciliella* Malloch, 1926 and *Viridinsula* Shannon, 1926. Their status needs assessment, and the results presented here suggest that morphological analyses alone will not be sufficient. Phylogenetic studies including a selection of both nuclear and mitochondrial genes are recommended.

***Hemipyrellia*** Townsend, 1918 (type species: *Lucilia fernandica* Macquart, 1855) was erected as a genus by Townsend (1918) and revised by Aubertin (1931). It had previously been suggested that *Hemipyrellia* was a synonym of *Lucilia* (Shannon 1926). *Hemipyrellia* is restricted to the Old World and the species are saprophagous. The results of this study place *Hemipyrellia* within *Lucilia* for both nuclear and mitochondrial analyses with 100% support (Figs 1 and 2), the *COI* barcode Bayesian tree (Fig. 5) with very strong support, and the morphological majority rule consensus tree (Fig. 6) with weak (56%) support.

In two studies of Australian blowflies, *Hemipyrellia* was found to be a sister-group to *Lucilia* (Wallman et al. 2005, Nelson et al. 2012), but these studies included only species of *Lucilia* that occur in Australia, thus *Hemipyrellia* may be a sister-clade to Australian *Lucilia* as an artefact of taxon sampling. Similarly, another study (Singh and Wells 2013) found *Hemipyrellia* to be sister-group to *Lucilia*, but this was based on one specimen of *Lucilia sericata* and one specimen of *Hemipyrellia fernandica*. Several other studies have sequenced *Hemipyrellia* specimens and found them to lie within *Lucilia* (Wells et al. 2007, Park et al. 2009, Liu et al. 2011, McDonagh and Stevens 2011). Two specimens of *Hemipyrellia ligurriens* from Taiwan (Fig. 2) group within the *Lucilia cuprina* clade. This is probably a misidentification because the specimens of *Hemipyrellia ligurriens* and *Hemipyrellia pulchra*, both from China, group with *Hemipyrellia fernandica* sequenced in this study. Assuming that the other *Hemipyrellia* specimens are not all misidentified, these previous studies together with the results of this study provide strong support for the synonymy of *Hemipyrellia* and *Lucilia*.

***Dyscritomyia*** Grimshaw, 1901 (type species: *Prosthetochaeta robusta* Grimshaw, 1901) contains 35 nominal species that are all found exclusively on the Hawaiian Islands (James 1981). The biology of *Dyscritomyia* differs from the other Luciliinae in that at least some species are viviparous and produce only one larva at a time that is retained in the uterus for the first two instar stages. Little is known about their parasitic behaviour but it is assumed that *Dyscritomyia* species are facultatively parasitic saprophages (Hardy 1981). *Dyscritomyia* was included in the *COI* Bayesian inference analysis and was recovered as a separate clade to *Lucilia* (Fig. 2). In previous studies, *Dyscritomyia* was recovered within *Lucilia* when analysing the *COI* and *EF-1α* genes (Wells et al. 2007, McDonagh and Stevens 2001) but it was recovered as a sister clade to *Lucilia* when analysing the *28S* gene (McDonagh and Stevens 2011). *Dyscritomyia* was also recovered as a sister group to *Lucilia* in a study of the *COI* and *COII* genes (Wells et al. 2002). The current study used only a 576 bp region of the total *COI* gene from the sequences available on GenBank that were used in the study of Wells et al. (2002), but still recovered *Dyscritomyia* as a sister clade to *Lucilia*. It therefore does not appear that the length of the *COI* sequence affects the analysis significantly.

This study used 20 species of *Lucilia* in the *COI* analysis while the previous studies used six and 13 species, respectively (Wells et al. 2002, McDonagh and Stevens 2011). The position of *Dyscritomyia* relative to *Lucilia* may be determined by the taxon sampling of *Lucilia*, as mentioned regarding *Hemipyrellia*. This highlights the need for a more comprehensive study of this genus and inclusion of as many *Dyscritomyia* and *Lucilia* species as possible to confirm the taxonomic relationship between *Dyscritomyia* and *Lucilia*.

***Hypopygiopsis*** Townsend, 1916 (type species: *Hypopygiopsis splendens* Townsend, 1916 = *Hypopygiopsis fumipennis* Walker, 1856) is restricted to the Asian and Australasian regions of the world (Kurahashi 1977). This genus apparently exhibits both oviparous and larviparous behaviour. The larval behaviour includes both facultative parasitism and saprophagy. *Hypopygiopsis* was included in the Bayesian inference analysis of the *COI* barcode dataset. One *Hypopygiopsis infumata* sequence grouped within *Lucilia* (Fig. 5) as part of a clade including *Lucilia hainanensis* + *Lucilia papuensis* + *Lucilia bazini*. On closer examination of the sequences, *Hypopygiopsis infumata* was identical to the *Lucilia bazini* sequence from China. The *Lucilia hainanensis* sequence from China that groups with these two sequences differs by only one base pair. This places doubt on the identification of these sequences and prevents any meaningful inferences being drawn. The second *Hypopygiopsis infumata* sequence groups with *Hemipyrellia*. There are only five sequences of *Hypopygiopsis* publically available and therefore the limited number of sequences constrains the credibility of this result and it is recommended that more sequences of this genus are examined to clarify if this genus should also be synonymised with *Lucilia*.

## Conclusion

*Lucilia sericata* and *Lucilia cuprina* are indeed sister-species. *Lucilia mexicana* is confirmed to be paraphyletic with respect to *Lucilia coeruleiviridis*, possibly as a result of hybridisation and introgression. *Lucilia caesar* and *Lucilia illustris* are both paraphyletic and further studies with different genes are needed to determine if these two species can be identified using molecular methods. *Hemipyrellia* should be synonymised with *Lucilia* because this genus sits within *Lucilia* in all of the analyses conducted in this study. *Dyscritomyia* requires further studies to confirm its phylogenetic positioning with regard to *Lucilia* because taxon sampling appears to have an impact on the analysis. The limited number of sequences available for *Hypopygiopsis* and the apparent misidentification of sequences prevent any conclusions being drawn about its relationship to *Lucilia*. In this study we have identified at least three cases of misidentified sequences from GenBank, which is a well-known problem (Bridge et al. 2003, Harris 2003, Nilsson et al. 2006, Valkiūnas et al. 2008). There is no geographic pattern to the distribution of the different parasitic behaviours within the Luciliinae and no reason to sub-divide *Lucilia* into genera or sub-genera based on either geographic location or parasitic behaviour.
